# Antiviral Efficacy of Lignan Derivatives (-)-Asarinin and Sesamin Against Foot-and-Mouth Disease Virus by Targeting RNA-Dependent RNA Polymerase (3D^pol^)

**DOI:** 10.3390/vetsci12100971

**Published:** 2025-10-10

**Authors:** Ploypailin Semkum, Natjira Mana, Varanya Lueangaramkul, Nantawan Phetcharat, Porntippa Lekcharoensuk, Sirin Theerawatanasirikul

**Affiliations:** 1 Department of Microbiology and Immunology, Faculty of Veterinary Medicine, Kasetsart University, Bangkok 10900, Thailand; fvetpls@ku.ac.th (P.S.); natjira.ma@ku.th (N.M.); fvetntw@ku.ac.th (N.P.); fvetptn@ku.ac.th (P.L.); 2 Department of Anatomy, Faculty of Veterinary Medicine, Kasetsart University, Bangkok 10900, Thailand; varanya.luea@ku.th; 3 Graduate Program in Animal Health and Biomedical Sciences, Faculty of Veterinary Medicine, Kasetsart University, Bangkok 10900, Thailand

**Keywords:** foot-and-mouth disease virus (FMDV), lignans, FMDV 3D^pol^, viral replication, antiviral activity

## Abstract

Foot-and-mouth disease (FMD) is a highly contagious viral infection affecting livestock. Although inactivated vaccines are commonly used, their effectiveness is limited by an immunity gap. Therefore, complementary antiviral strategies are required for effective control and prevention. Lignans, plant-derived compounds, have shown promising antiviral properties, yet their potential against foot-and-mouth disease virus (FMDV) remains underexplored. This study employed virtual screening to identify lignan compounds targeting viral RNA-dependent RNA polymerase (3D^pol^). Six lignan compounds were selected for cytotoxicity and antiviral activity evaluation including pre-viral entry, post-viral entry, and protective effect assays. Antiviral activity assay showed that (-)-asarinin and sesamin exhibit potent inhibition effects in the post-viral entry with EC50 of 15.11 μM and 52.98 μM, respectively, using immunoperoxidase monolayer assay. Both compounds exhibited dose-dependent reduction in viral replication with significant suppression of negative-strand RNA production. Lignans’ ability to target FMDV 3D^pol^ was further confirmed using a cell-based FMDV minigenome assay. Among the tested lignans, (-)-asarinin demonstrated remarkable inhibition of GFP expression (IC50 value at 10.37 μM), while sesamin required a higher concentration for similar effects. *In silico* prediction revealed that these lignans preferentially bind to FMDV 3D^pol^ active site. These findings are the first to establish (-)-asarinin and sesamin as promising antiviral candidates against FMDV.

## Simple Summary

Foot-and-mouth disease (FMD) is a highly contagious viral disease that severely impacts livestock worldwide, causing major economic losses. Our research explored new ways to combat this virus using natural compounds. We investigated two plant-derived compounds called (-)-asarinin and sesamin and discovered that they are highly effective at stopping the FMD virus in cell culture. In our study, we found that these two compounds work by targeting and disabling a key part of the virus’s machinery, specifically an enzyme it needs to replicate itself. By blocking this “copying machine” (known as 3D^pol^), (-)-asarinin and sesamin prevent the virus from multiplying and spreading. This discovery represents a significant step toward developing new and effective antiviral treatments that could help protect livestock and control the spread of foot-and-mouth disease.

## 1. Introduction

Foot-and-mouth disease (FMD) is a severe, highly contagious viral disease affecting cloven-hoofed animals, including cattle, swine, sheep, and goats [1,2]. This transboundary disease significantly impacts livestock production and international trade, with clinical manifestations including fever, blisters, and lameness [1,2,3]. The causative agent, Foot-and-Mouth Disease Virus (FMDV), belongs to the genus *Aphthovirus* within the *Picornaviridae* family [3,4]. Seven viral serotypes exist (A, O, SAT1, SAT2, SAT3, and Asia1), though serotype C has not been reported since 2004 [5]. Current control strategies face significant challenges, including serotype-specific vaccines with limited cross-protection [2,4], difficulties differentiating vaccinated from infected animals, and requirements for animal culling. Antivirals and biotherapeutics have been suggested as critical interventions, offering rapid protection before vaccine-induced immunity develops and potentially mitigating the severe economic and agricultural impacts of the disease [6,7].

FMDV contains a single positive-sense RNA genome encoding a polyprotein that undergoes proteolytic processing to yield structural and non-structural proteins. The 3D protein (3D^pol^) functions as RNA-dependent RNA polymerase, crucial for viral replication, with a structure featuring thumb, fingers, and palm subdomains that form the catalytic site [8,9,10]. Being highly conserved across all FMDV serotypes [10,11], 3D^pol^ represents an ideal target for broad-spectrum antivirals.

Natural products, particularly lignans, are promising candidates for antiviral drug development due to their diverse biological activities and established safety profiles. These plant-derived polyphenolic compounds have demonstrated broad-spectrum antiviral properties against various viral pathogens [12,13], yet have not been previously investigated as inhibitors of FMDV. This study represents the first systematic exploration of lignans as potential anti-FMDV agents, paving the way for antiviral development against this economically significant pathogen.

This research employs an integrated approach combining computational screening with experimental validation to identify FMDV inhibitors from natural lignans. Our workflow utilizes virtual screening to identify promising candidates targeting the 3D^pol^ enzyme, followed by a suite of cell-based studies to confirm their efficacy and mechanism. The primary objective of this study is to be the first to investigate and validate the lignan chemical scaffold, previously unexplored for this purpose, as a novel class of FMDV 3D^pol^ inhibitors. By identifying and validating active compounds, this work aims to open a new avenue for the development of novel antiviral therapeutics, ultimately supporting existing FMDV control strategies.

## 2. Materials and Methods

### 2.1. Virtual Screening of Lignan Compounds and Molecular Docking

The crystal structure of FMDV 3D^pol^ was retrieved from a reference 3D^pol^ (PDB code ID: 1wne.pdb [9] and used as a template for modeling of FMDV serotype A ((A/TAI/NP05/2017); NP05) as a macromolecule 3D structure, which was described previously [14,15]. A comprehensive lignan compound library was assembled from multiple sources: 82 compounds from the Plant Secondary Compounds (PSC) database [16] and 381 compounds from ChemFaces (Wuhan, China). The 3D structures of these compounds were retrieved from PubChem (https://pubchem.ncbi.nlm.nih.gov, accessed on 1 October 2024). All compounds were initially screened for ADME/Tox properties using SwissADME software (http://www.swissadme.ch/, accessed on 29 October 2024) [17].

**Figure 1:**
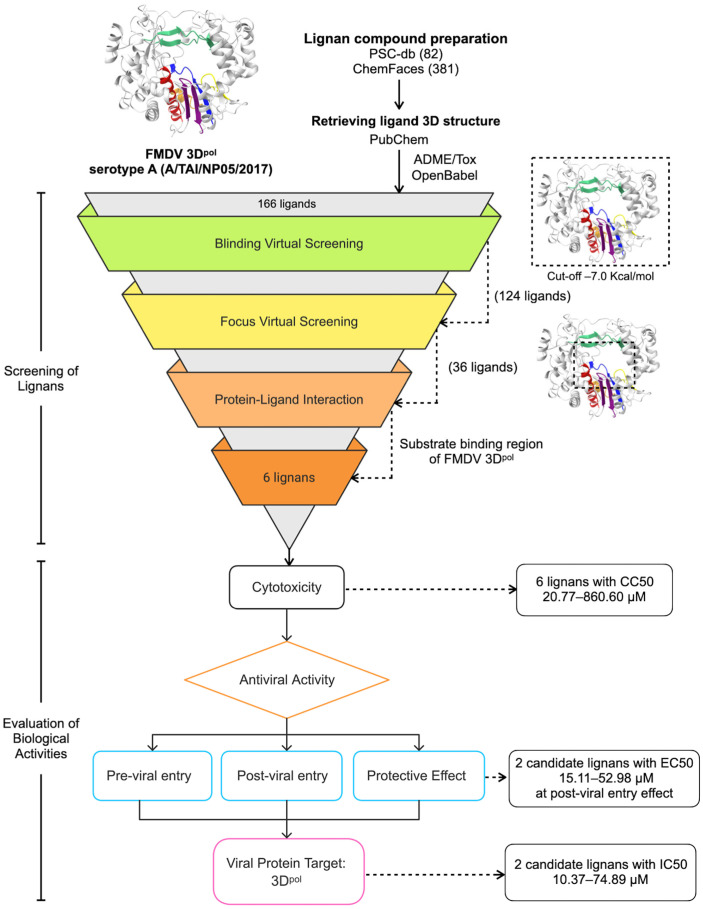
Experimental workflow for identifying lignan compounds targeting FMDV 3D^pol^. FMDV 3D^pol^ structure shown with Motifs A–F in different colors. Screening process: 166 commercially available lignan compounds from ligand databases were assessed for predicted ADME properties. Blind virtual screening and focused docking to the 3D^pol^ active site were performed, followed by cell-based assays to confirm antiviral activity of the top candidate lignans.

The virtual screening process followed a two-step approach as illustrated in Figure 1. First, blind virtual screening was conducted where the grid box covered the entire FMDV 3D^pol^ molecule. The molecular docking procedure employed AutoDock Vina [18], facilitated by PyRx software version 0.9.8 [19]. Docking parameters were set with an exhaustiveness of 20 and a maximum number of binding modes set to 9. The docking process employed an empirical scoring function to evaluate ligand-protein interactions. Grid parameters were defined with coordinates provided of X = 19.34, Y = 31.20, Z = 23.25, within a grid box size of 65 Å × 70 Å × 65 Å [14].

In the second step, focused docking was performed specifically targeting the substrate binding region Motifs A-F of FMDV 3D^pol^ [9]. This included the palm subdomains (Motif A and C) of FMDV 3D^pol^, encompassing the conserved amino acid residues crucial for its functionality, including Asp240, Asp245 (motif A), Asp338, and Asp339 (motif C) of FMDV 3D^pol^ [9,10,11]. Additionally, amino acid residues Pro44, Pro169, and Met296 were also included for analysis. The grid parameters for focused docking were set to target the binding pocket containing the catalytic residues within motifs A–F, with a grid center positioned at X = 15, Y = 26, Z = 15 and dimensions of 30 Å × 30 Å × 35 Å. Docking outcomes were analyzed through molecular visualization of protein–ligand interactions using Discovery Studio Visualizer, version 2021 (BIOVIA, Dassault Systèmes, San Diego, CA, USA), and UCSF ChimeraX, version 0.94 (UCSF, San Francisco, CA, USA) [20]. The virtual screening and selection process is depicted in the workflow in Figure 1. Based on the virtual screening results, six commercially available lignan compounds were selected and purchased for further biological evaluation.

### 2.2. Cells, Viruses, and Lignans

BHK-21 cells (ATCC^®^, Manassas, VA, USA) at passages 16–25 were used for all cell-based assays. The cells were cultured in complete medium containing MEM (Invitrogen^TM^, Carlsbad, CA, USA), 10% FBS, 2 mM L-glutamine, and 1 × Antibiotic-Antimycotic at 37 °C with 5% CO_2_. FMDV serotype A (A/TAI/NP05/2017; NP05) [21] was propagated in BHK-21 cells for 24 h to generate virus stock. After the sixth passage of viral propagation, the half tissue culture infective dose (TCID50) was calculated according to the Reed–Muench method [22], yielding a titer of 1 × 10^7^ TCID50/mL. The virus stock was preserved at −80 °C in single-use aliquots. All procedures with live FMDV were conducted in a biosafety level-2 facility with enhanced safety measures.

Selected lignan compounds from virtual screening were obtained from ChemFaces (Wuhan, China) and prepared as 10 mM stock solutions in Dimethyl sulfoxide (DMSO, Sigma-Aldrich, St. Louis, MO, USA). These compounds were stored at –20 °C for subsequent *in vitro* cell-based experiments. Ribavirin (Sigma Aldrich, St. Louis, MO, USA) served as the drug control in all experiments.

### 2.3. Cytotoxicity Assay

Cytotoxicity of lignans was evaluated using Cell Counting Kit-8 (CCK-8, TargetMol^®^, Wellesley Hills, MA, USA) in BHK-21 cells. Cells were seeded at 2 × 10^4^ cells per well in 96-well plates and incubated at 37 °C with 5% CO_2_ overnight. Lignan compounds were diluted in serum-free media containing 0.01% DMSO. Serum-free media with and without DMSO served as controls. The diluted compounds (100 µL) were added to each well and incubated at 37 °C for 24 h. CCK-8 solution (10 µL) was then added to each well and incubated for 2 h at 37 °C [14]. Absorbance was measured at 450 nm using a Synergy H1 Hybrid Multi-Mode Reader (BioTek^®^, Winooski, VT, USA). Cell viability percentage was calculated as previously described [14], and cytotoxic concentration 50% (CC50 values) were determined by using GraphPad Prism, version 8.4.0.

### 2.4. Antiviral Activity Assay

BHK-21 cells were seeded at 2 × 10^4^ cells per well in 96-well plates and grown overnight through incubation at 37 °C with 5% CO_2_. Cells were inoculated with FMDV at a multiplicity of infection (MOI of 0.0002) diluted in complete media. The antiviral activity of lignans was evaluated through pre-viral entry, post-viral entry, and protective effect assays [14,23]. Ribavirin and DMSO vehicle served as positive and negative drug control. For the pre-viral entry assay, each lignan was incubated with the virus inoculum at 37 °C for 2 h, the mixture was removed, and cells were incubated with 100 µL complete media for 24 h. For the post-viral entry assay, the virus inoculum was incubated with cells at 37 °C for 2 h, the spent inoculum was replaced with 100 µL complete media, and cells were treated with lignans for 24 h. For the protective effect, cells were pre-treated with lignans at 37 °C for 2 h, then the virus inoculum was added for 2 h, followed by replacement with fresh complete media and incubation at 37 °C for 24 h. After each experiment, cells were fixed and stained with 0.5% crystal violet solution and evaluated using the immunoperoxidase Monolayer Assay (IPMA) for antiviral activity using a phase-contrast inverted microscope (Olympus IX73, Tokyo, Japan) at 200 × magnification [14,23].

### 2.5. Immunoperoxidase Monolayer Assay (IPMA)

BHK-21 cells grown overnight in a 96-well plate were inoculated with FMDV serotype A (NP05) and treated with diluted lignan compounds. DMSO-treated and ribavirin-treated cells were used as antiviral drug negative and positive controls, respectively. At 24 hpi, cells were fixed with cold methanol for 15 min at room temperature and washed three times with 1× phosphate-saline buffer containing 0.05% Tween20 (PBST, Sigma Aldrich^®^, St. Louis, MO, USA). Cells were treated with BlockPRO™ 1 Min Protein-free Blocking Buffer (Taipei, Taiwan) for 1 min, followed by three 1× PBST washes. The cells were then incubated at 37 °C for 1 h with primary antibody (scFv-Fc specific to FMDV 3ABC proteins [24,25] at 1: 200 dilution. After three 1×PBST washes, cells were incubated with secondary antibody (HRP-conjugated protein G, EMD Millipore Corporation, Temecula, CA, USA) at 1: 500 dilution at 37 °C for 1 h. Following three washes with 1× PBST, cells were incubated with DAB substrate (DAKO, Santa Clara, CA, USA).

Dark-brown intracytoplasmic staining indicative of FMDV-infected cells was recorded using a phase-contrast inverted microscope (Olympus IX73, Tokyo, Japan) at 200× magnification. Cell images were analyzed using CellProfiler image analysis v.4.2.6 to quantify the number of positively infected cells. Cell morphology was observed using the same microscope with a 20× objective. Images were captured using an Olympus IX73 digital camera with CellSens Standard software (version 3.1) at a resolution of 1920 × 1080 pixels. Five random fields per well were imaged using fixed exposure settings (300 ms exposure time). Cell analysis was performed using CellProfiler with the following parameters: RGB color-based input raw image, primary object identification (typical diameter range: 1–10,000 pixels), background correction using the “Regular” method, and intensity thresholding using the manual method at 0.1–0.4. The 50% effective concentration (EC50) was determined using GraphPad Prism, version 8.4.0, as previously described [14].

The resulting data were utilized to compute the EC50, following previously described methods [23,24]. This value represents the concentration of the compound at which viral infection was reduced by 50% compared to the DMSO control. The analysis consisted of establishing the DMSO control as 100% infection and determining the EC50 value for each compound utilizing GraphPad Software version 8.4.0 (Prism, San Diego, CA, USA).

### 2.6. Viral Copy Number Quantification Using RT-qPCR

BHK-21 cells were seeded at 2 × 10^5^ cells per well in 24-well plates (Corning Incorporated., Corning, NY, USA) and cultured overnight. Cells were incubated with FMDV serotype A (NP05, MOI = 0.0002) for 2 h, followed by removal of viral inoculum. Infected cells were treated with serially diluted lignan compounds and incubated at 37 °C for an additional 22 h. Total RNA was extracted using Direct-zol^TM^ RNA MiniPrep (Zymo Research Corporation, Tustin, CA, USA) according to the manufacturer’s instructions. RNA quantity and purity were assessed using a NanoDrop^TM^ 2000c Spectrophotometer (Thermo Fisher Scientific, Waltham, MA, USA). Viral RNA was reverse transcribed using either random hexamer primers (Invitrogen^TM^, Carlsbad, CA, USA) or a gene-specific primer (Table S1) targeting the 3D^pol^ coding region for negative-strand RNA detection [14,23]. cDNA synthesis was performed using RevertAid reverse transcriptase (Thermo Fisher Scientific Inc., Waltham, MA, USA) following the manufacturer’s instructions.

qPCR was performed using the synthesized cDNAs and iTaq™ Universal SYBR^®^ Green Supermix (Bio-Rad Laboratories, Hercules, CA, USA) in the CFX96 Touch thermal cycler (Bio-Rad Laboratories, USA). The reaction contained 5 µL iTaq™ Universal SYBR^®^ Green Supermix and 0.5 µL of each target-specific forward and reverse primer (Table S1) (FMDV-5′UTR_F and FMDV-5′UTR_R for the random hexamer-derived cDNA or FMDV-3D_F and FMDV-3D_R for cDNA derived from gene-specific primer reaction) [14,21,23], 2 µL cDNA, and nuclease-free ddH_2_O to 10 µL. The amplification protocol consisted of initial denaturation at 95 °C for 30 sec, followed by 40 cycles of 95 °C for 5 sec and 60 °C for viral load quantification or 55 °C for negative-stranded RNA quantification for 30 sec. Melting curve analysis was performed from 65 °C to 95 °C with a 0.5 °C increment. Viral RNA levels were determined by absolute quantification using a standard curve generated from 10-fold serial dilutions (10^−2^ to 10^−7^ plasmid molecules/µL) of plasmid containing FMDV 5′ UTR [14,21,23]. Negative-stranded RNA synthesis was quantified using delta Ct values (difference between Ct values of compound-treated and untreated FMDV-infected cells). Normalized delta Ct was calculated as the following equation [14,21,23] and displayed as a percentage of the normalized delta Ct. Data from three independent experiments are presented as means ± SD using GraphPad Prism version 8.4.0 (Prism, San Diego, CA, USA).

### 2.7. Evaluation of 3D^pol^ Inhibition Activity Using a Cell-Based FMDV Minigenome Assay

The FMDV minigenome assay was performed to assess the inhibitory effects of lignan compounds on FMDV 3D^pol^, which is an essential viral protein involved in viral replication. The assay utilized pKLS3_GFP, a plasmid that carried the enhanced green fluorescent protein (GFP) gene inserted between FMDV 5′ and 3′UTRs (cis-acting elements for FMDV transcription and translation), along with two helper plasmids: pCAGGS_T7 encoding T7 RNA polymerase, and pCAGGS_P3 encoding the FMDV P3 proteins [14,21,23]. BHK-21 cells were plated at 2 × 10^4^ cells per well in a 96-well plate and incubated overnight at 37 °C. The three plasmids were mixed with Fugene^®^ HD (Promega, Madison, WI, USA) and Opti-MEM™ Reduced-Serum Medium (Gibco^TM^ Thermo Fisher Scientific Inc., Waltham, MA, USA) to achieve a final volume of 10 µL per well. After 15-min incubation at room temperature, the transfection mixture was substituted with serially diluted lignan compounds in Opti-MEM™. Controls included a vehicle control (0.01% DMSO) and a plasmid control (pKLS3_GFP without helper plasmids), and the transfected cells were maintained at 37 °C with 5% CO_2_. GFP expression was assessed at 24 h post-transfection (hpt). Fluorescent signals were quantified using CellProfiler image analysis software (v.4.2.6) to determine the number of positively infected cells. Cell morphology was observed using a 20× objective, and images were captured as mentioned above. Image analysis was performed using CellProfiler with the following parameters: GFP-based input raw image, grayscale output image, relative weight to red/blue/green channels of 1, primary object identification (typical diameter range: 10–250 pixels), and intensity thresholding using the Otsu method with a correction factor of 0.6. The half-maximal inhibitory concentration (IC50) was determined using GraphPad Prism (v.8.4.0) as previously described [14,21,23]. The IC50 represents the concentration at which 3D^pol^ activity was reduced by 50% compared to that of the DMSO control.

### 2.8. Intracellular FMDV 3C^pro^ Inhibition Assay

A cell-based FMDV 3C protease inhibition assay was established using a transcriptional reporter system, adapted from previously described protocols [26,27]. The assay employed three key components: (1) pBV_3ABCD encoding active FMDV 3C^pro^ fused with Gal4-binding domain and VP16 activation domain, (2) pBV_mu3ABCD containing inactive 3C^pro^ (Cys142Ser and Cys163Gly mutations) as a negative control, and (3) pG5Luc reporter plasmid containing firefly luciferase under Gal4/VP16 regulation. Both 3C^pro^-expressing plasmids incorporated Renilla luciferase as an internal control.

HEK-293T cells maintained in Opti-MEM were seeded in 96-well plates (2 × 10^5^ cells/well) and incubated overnight at 37 °C with 5% CO_2_. Cells were co-transfected with either pBV_3ABCD or pBV_mu3ABCD (100 ng) plus pG5Luc (100 ng) using Fugene^®^ HD (0.6 μL/μg DNA) in duplicate wells per condition, following manufacturer’s recommendations [26,27]. After 2 h, the medium was replaced with fresh Opti-MEM containing serial dilutions of test compounds or 0.1% DMSO vehicle control. Rupintrivir, a known 3C^pro^ inhibitor [26,27], served as a positive control. Following 16 h compound exposure, cells were washed with PBS, lysed with 20 μL passive lysis buffer, and luminescence was measured using the Dual-Glo Luciferase Assay System on a Synergy H1 reader. The assay principle relies on 3C^pro^-mediated cleavage of the fusion protein, which prevents transcriptional activation of firefly luciferase, as previously demonstrated [26,27]. Effective 3C^pro^ inhibitors preserve the fusion protein integrity, resulting in increased firefly luciferase expression. Inhibitory activity was quantified as the ratio of firefly to Renilla luciferase signals (Fluc/Rluc) compared to controls, using established methods [26,27]. Protease activity was reported as an inverse correlation of the Fluc/Rluc ratio from compound-treated wells relative to no-drug control.

### 2.9. Statistical Analysis

All experiments were performed with two biological replicates, each with technical duplicates, and repeated in three independent experiments. The half maximum cytotoxic concentration (CC50), maximum non-toxic concentration (CC10), half maximum effective concentration (EC50), and half maximum inhibitory concentration (IC50) were calculated using non-linear regression with variable slope (four parameters) with 95% confidence intervals. Data are presented as the mean ± standard deviation (SD). Statistical differences between test conditions and controls were determined using one-way ANOVA followed by Tukey’s multiple comparisons test (GraphPad Prism version 8.4.0), with *p* < 0.05 considered statistically significant.

## 3. Results

### 3.1. Virtual Screening

Virtual screening identified lignans with potential inhibitory activity against FMDV 3D^pol^ (Figure 1). Our initial selection focused on 166 commercially available lignans that passed favorable ADMET property screening to ensure their suitability for subsequent *in vitro* testing. Blind virtual screening further filtered these to 124 lignans using a binding energy cutoff of –7.0 kcal/mol. Focused docking to the FMDV 3D^pol^ active site subsequently identified 36 lignans that effectively bound to the enzyme’s active sites. Final selection yielded six lignans: (-)-asarinin, paulownin, sesamin, eleutheroside E, gomisin A, and picropodophyllotoxin. These compounds preferentially interacted with active domain residues of FMDV 3D^pol^, including Asp240, Asp245, Asp338, and Asp339 (Figure S1). Detailed virtual screening results and ADME/Tox properties are provided in Table S2 and Table S3. These six selected lignans were advanced for experimental validation.

### 3.2. Cytotoxicity of Lignans on BHK-21 Cells

**Figure 2:**
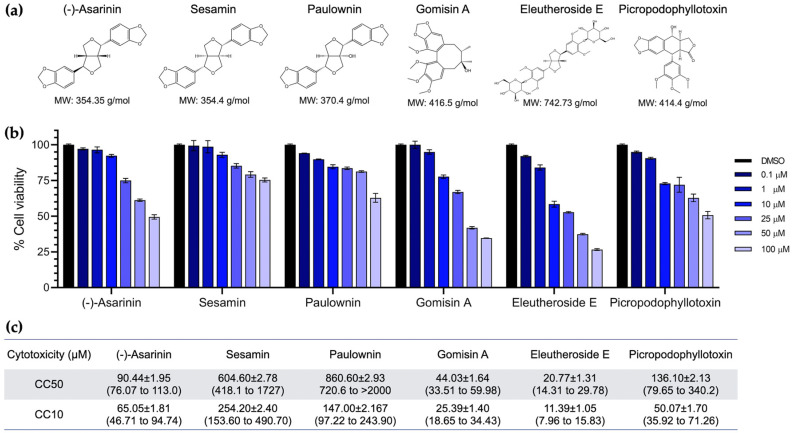
Chemical structures and cytotoxicity profiles of selected lignan compounds. (**a**) Six top-ranked lignan compounds identified through virtual screening. (**b**) Cell viability assessed using the CCK-8 assay, expressed as percentage viability at various compound concentrations. (**c**) The CC50 and CC10 values of selected lignan compounds with a 95% confidence interval.

To exclude the possibility that antiviral activity was due to compound cytotoxicity, the effects of six lignan compounds on BHK-21 cells were determined using the CCK-8 assay (Figure 2a). Cells were treated with lignans at concentrations of 0.1, 1, 10, 25, 50, and 100 µM. Among the six lignan compounds tested, paulownin and sesamin exhibited mild toxicity, maintaining cell viability at relatively high concentration (100 µM), while (-)-asarinin, eleutheroside E, gomisin A, and picropodophyllotoxin showed moderate cytotoxicity at the same concentration (Figure 2b,c). The CC50 and CC10 values are presented in Figure 2c. Compounds demonstrating mild-to-moderate toxic effects on BHK-21 cells were subsequently tested for antiviral activity in a dose–response manner, with testing concentrations based on the established cytotoxicity profiles.

### 3.3. (-)-Asarinin and Sesamin Inhibit Cytopathic Effects After FMDV Infection in BHK-21 Cells

**Figure 3:**
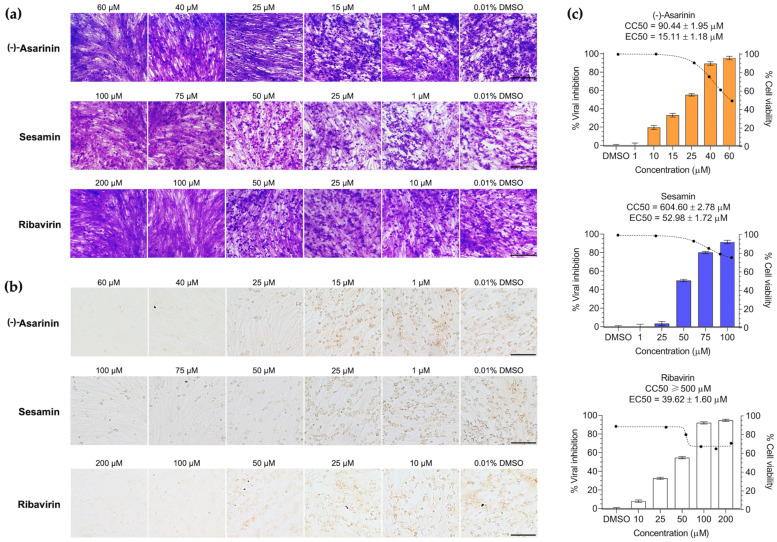
Cell-based antiviral activity of selected lignan compounds against FMDV after infection in BHK-21 cells. (**a**) Crystal violet staining showing morphological changes in FMDV-infected BHK-21 cells, from spindle-shaped to rounded cells. (**b**) Immunoperoxidase monolayer assay detecting FMDV antigens in infected cell cytoplasm. (-)-Asarinin and sesamin reduced the number of FMDV-positive cells dose-dependently. Ribavirin and DMSO served as positive and vehicle controls, respectively. Scale bars: 200 μm. (**c**) Dose–response graphs of viral inhibition (bars) and cell viability (dotted line), with EC50 and CC50 values.

Six lignan compounds were investigated for their antiviral activity at three stages of FMDV infection, including pre-viral entry, post-viral entry, and protective effect. In the post-viral entry assay, two of the six lignans, (-)-asarinin and sesamin, demonstrated dose-dependent inhibition of FMDV infection in treated cells, as shown in Figure 3a. These compounds reduced cell rounding, cluster formation, and aggregation, as visualized through crystal violet staining. The remaining compounds showed no inhibition compared to the ribavirin control. Neither the pre-viral entry nor protective effect assays showed inhibition by any of the six lignans (Figure S2). Consequently, (-)-asarinin and sesamin were selected for further testing on viral protein expression and FMDV replication in BHK-21 cells.

### 3.4. (-)-Asarinin and Sesamin Reduce FMDV Protein Expression and Replication in a Dose-Dependent Manner

**Figure 4:**
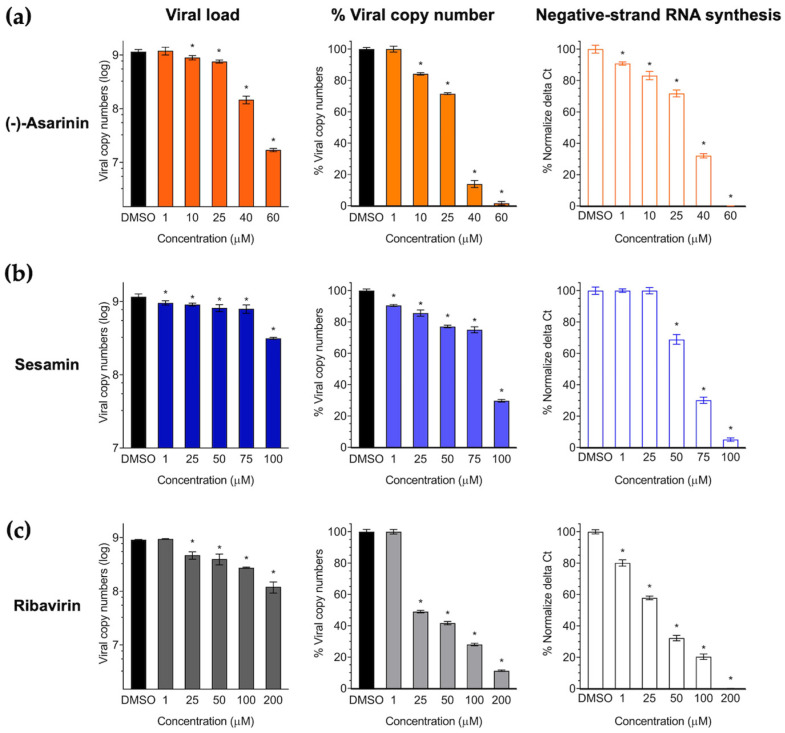
Antiviral effects of (-)-asarinin and sesamin on FMDV RNA production in BHK-21 cells. (**a**) (-)-asarinin, (**b**) sesamin, and (**c**) ribavirin (drug control). Viral load reduction is shown as viral copy number (left), and percentage of viral copy number (middle). Reduction of negative-strand RNA synthesis is shown by normalized ∆Ct values (right). Asterisk (*) indicates statistical significance (*p* < 0.001).

Subsequently, the initial antiviral activities of lignans (-)-asarinin and sesamin were further investigated. Their efficacy against FMDV infection in BHK-21 cells was assessed using the IPMA method, which demonstrated a significant and dose-dependent reduction in the number of positively infected cells (brown-stained) (Figure 3b,c). The EC50 values were 15.11 ± 1.18 μM (95% CI: 13.72 to 16.49) for (-)-asarinin and 52.98 ± 1.72 μM (95% CI: 51.60 to 54.36) for sesamin. Notably, (-)-asarinin exhibited more than 2.5-fold greater potency than the reference antiviral drug ribavirin (EC_50_ = 39.62 ± 1.60 μM, 95% CI: 38.34 to 40.90) under identical assay conditions (Table 1). These findings were consistent with the replication analysis using RT-qPCR, which quantified viral loads by detecting the 5′ untranslated region (5′ UTR) of FMDV and measuring negative-strand RNA synthesis. For (-)-asarinin, concentrations of 1, 10, 25, 40, and 60 µM were tested, while for sesamin, concentrations of 1, 25, 50, 75, and 100 µM were examined (Figure 4). The results demonstrated a robust dose-dependent reduction in viral copy numbers with both compounds. In particular, (-)-asarinin completely inhibited 50% of FMDV at 40 μM, while sesamin showed markedly significant inhibition at a concentration of 100 μM. Furthermore, we examined the effects of the compounds on negative-stranded RNA synthesis. Our findings revealed that (-)-asarinin achieved remarkable inhibition (>60%) at a concentration of 40 µM of negative-strand RNA production mediated by 3D^pol^, confirming its efficacy as an FMDV 3D^pol^ inhibitor (Table 1 and Figure 4). These findings support the potential of these compounds, particularly (-)-asarinin, as promising candidates with antiviral activities targeting viral RNA synthesis.

### 3.5. (-)-Asarinin and Sesamin Potentially Affect FMDV 3D^pol^ Activity in Transfected BHK-21 Cells

**Figure 5:**
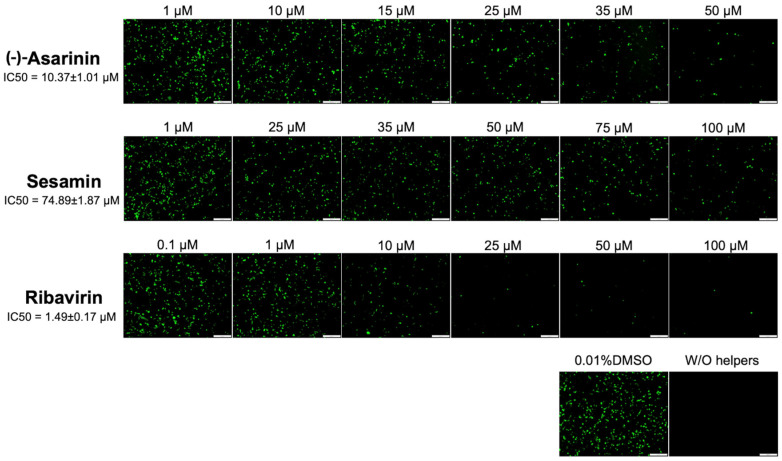
Inhibitory effects on FMDV 3D^pol^ activity using the minigenome screening assay. GFP expression relates to FMDV 3D^pol^ activity. (-)-Asarinin and sesamin were evaluated dose-dependently, with ribavirin as a positive control. Controls included DMSO (vehicle) and pKLS3_GFP without helper plasmids. Scale bars: 200 μm.

According to virtual screening targeting FMDV 3D^pol^ and the antiviral activity of selected lignans, we further explored the mechanism by which the lignan compounds inhibited FMDV 3D^pol^ activity using a cell-based FMDV minigenome assay as previously described [14,21,23]. This assay served as an effective screening tool to evaluate the inhibition of FMDV 3D^pol^ activity, which relates to the reduction in GFP intensity and the number of positive cells in compound-treated wells compared to the negative drug control wells. The IC50 values of the lignan compounds against 3D^pol^ activity were determined. Notably, (-)-asarinin demonstrated remarkable inhibitory activity with an IC50 of 10.37 ± 1.01 µM (95% CI: 9.56 to 11.18), exhibiting potency comparable to the reference compound ribavirin (IC50 = 1.49 ± 0.17 µM; 95% CI: 1.35 to 1.63). Sesamin showed inhibitory effects with an IC50 value of 74.89 ± 1.87 µM (95% CI: 73.39 to 76.39) (Table 1 and Figure 5). We further investigated other viral proteins, such as FMDV 3C^pro^, through inhibitory assays (Figure S3). We found that these lignans did not affect FMDV 3C^pro^ activity, suggesting their specificity for 3D^pol^ inhibition. These findings provided compelling evidence that both lignan compounds effectively reduce 3D^pol^ function, with (-)-asarinin showing particularly high potency, while sesamin also demonstrated notable inhibitory activity at higher concentrations (Table 1 and Figure 5).

### 3.6. Molecular Docking Confirms Specific Binding Interactions Between Lignans and FMDV 3D^pol^ Catalytic Domains

**Figure 6:**
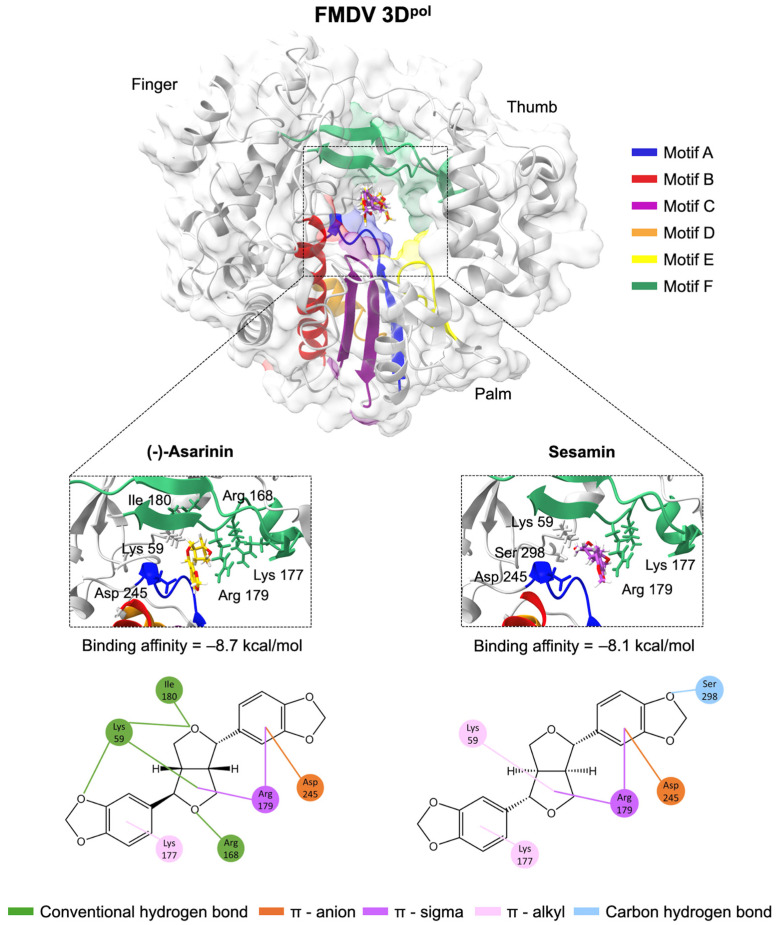
Lignan interactions with FMDV 3D^pol^ binding sites. Protein–ligand interactions of (-)-asarinin (yellow) and sesamin (purple) occupying motifs A (blue) and F (green), associated with catalytic residue Asp245. Three-dimensional (upper) and two-dimensional (lower) interaction structures.

To further understand the molecular basis for the observed 3D^pol^ inhibitory activity, we confirmed the potential interactions of the lignans with FMDV 3D^pol^ as depicted in Figure 6. Both (-)-asarinin and sesamin share a common tetrahydro-1H,3H-furo [3,4-c]furan core with 1,3-benzodioxole substituents but differ in their stereochemistry and positioning of the benzodioxole groups. Molecular docking results revealed that the 1,3-benzodioxole groups of (-)-asarinin and sesamin could interact with the catalytic residue Asp245 of motif A through π-anion interactions. The motif F of the finger subdomain, which contains additional triphosphate group residues Arg168, Lys177, and Arg179 [9,10,11], demonstrated that Arg179 interacted with the core structure and 1,3-benzodioxole groups of both lignans via π-sigma interactions. More importantly, (-)-asarinin showed an additional interaction between the oxygen atom of its core structure and Arg168, Ile180, and Lys59 through conventional hydrogen bonding. These molecular interactions provide a structural basis for the stronger inhibitory activity observed with (-)-asarinin (IC50 = 10.37 ± 1.01 µM) compared to sesamin (IC50 = 74.89 ± 1.87 µM) (Table 1) in the cell-based 3D^pol^ assay. Consistently, molecular docking analysis revealed a more favorable binding affinity for (-)-asarinin (−8.7 kcal/mol) compared to sesamin (−8.1 kcal/mol), further supporting its stronger interaction with FMDV 3D^pol^.

## 4. Discussion

In this study, we identified (-)-asarinin and sesamin as promising inhibitors of FMDV 3D^pol^ through virtual screening and confirmed their antiviral activity through multiple experimental approaches. Our study is the first to demonstrate the efficacy of these lignans against a picornavirus polymerase, particularly FMDV 3D^pol^. These furofuran-type lignans have distinct sources: sesamin is the major lignan constituent from *Sesamum indicum* seeds and sesame oil, while (-)-asarinin, an epimer of sesamin, is derived from *Asarum* spp., particularly *Asarum sieboldii* Miq., commonly used in traditional Chinese medicine [12,13,28,29]. Both compounds have been shown to possess antioxidant, anti-inflammatory, and anticancer activities [12,13]. Notably, we observed that the effect of (-)-asarinin against FMDV infection (15.11–36.70 μM) is comparable to that reported for a nucleoside inhibitor (NI), ribavirin (28.09 µM in IBRS-2 cells; 40.92 ± 1.61 µM in BHK-21 cells) against FMDV in previous studies [23,30], suggesting similar potency to this established antiviral.

Our findings extend previous research on these compounds’ antiviral properties against other viruses. Sesamin (at 5 µg/mL) has been reported to inhibit influenza A (H1N1) virus by reducing the concentration of inflammatory cytokines in human PBMCs and inhibited influenza virus neuraminidase without exhibiting cytotoxicity [31]. An *in silico* study has shown asarinin’s potential binding to viral protease, SARS-CoV-2 3CL^pro^ [32]. Neither sesamin nor (-)-asarinin inhibited FMDV 3C^pro^ in our study, indicating specificity in their antiviral mechanism. These results align with previous studies on other lignan-derived RdRp inhibitors, such as silymarin components against hepatitis C virus NS5B RdRp activity at concentrations ranging from 74.5 ± 5.5 µM to 97.2 ± 24.1 µM [33]. An *in silico* study demonstrated (-)-asarinin’s potential against Zika virus methyltransferase and Zika RNA-dependent RNA polymerase [34].

Time-of-addition studies revealed that these lignans primarily exert their antiviral effects during the post-entry phase of viral replication, supporting our conclusion that 3D^pol^ inhibition is the primary mechanism of action. Structure–activity relationship analysis showed that despite sharing an identical tetrahydro-1H,3H-furo [3,4-c]furan core with two 1,3-benzodioxole substituents, their distinct spatial configurations significantly influence inhibitory activity. (-)-Asarinin exhibited approximately 7-fold greater potency (IC50 = 10.37 ± 1.01 µM) compared to sesamin (IC50 = 74.89 ± 1.87 µM), suggesting that the trans configuration of the benzodioxole groups creates optimal interactions with the polymerase’s active site. The superior molecular interaction between (-)-asarinin and the 3D^pol^ active site over sesamin strengthens its higher inhibitory effect on FMDV in all cell-based assays. This finding aligns with previous studies on HIV reverse transcriptase inhibitors, where subtle stereochemical differences led to substantial variations in antiviral efficacy [35].

Molecular docking analyses revealed that both compounds interact with the catalytic residue Asp245 of motif A through π-anion interactions via the 1,3-benzodioxole groups of lignans, similar to the binding of known nucleotide analog inhibitors such as ribavirin triphosphate, as shown in a previous study (PDB: 2E9R [34]). In addition, the furo [3,4-c]furan core of both lignans also interacted with residues Arg168 and Arg179, which play an essential role in NTP substrate binding [36]. This corresponded with the finding that the oxygen atom of this core formed hydrogen bonds with conserved arginine residues, enhancing binding affinity with H1N1 neuraminidase [31]. To our current understanding, this interaction likely stabilizes (-)-asarinin within the active site, enhancing its inhibitory effect by interfering with the rNTP substrate selection process, which is essential for 3D^pol^ function and initiating the uridylylated form of VPg to 3D^pol^ [9,10,11]. Based on these interaction patterns, (-)-asarinin represents a promising candidate that functions as a non-nucleoside inhibitor (NNI) of FMDV 3D^pol^ [14,37,38] with superior binding characteristics compared to sesamin.

Our *in silico* screening also examined four additional compounds (Figure S1). Paulownin and picropodophyllotoxin did not form sufficiently strong bonds with key catalytic residues, while gomisin A and eleutheroside E interacted with Asp338 and Asp339 but showed no potent effect in our cell-based assay. These results suggest that the orientation of the 1,3-benzodioxole groups of lignans relative to the catalytic site is a key determinant of activity.

The limitations of serotype-specific vaccines in controlling FMD outbreaks highlight a critical need for broad-spectrum, direct-acting antivirals. The potent activity of (-)-asarinin suggests its potential clinical application as a therapeutic or prophylactic agent capable of being deployed rapidly to mitigate disease spread during an outbreak, regardless of the circulating serotype. This positions our findings as a crucial first step towards developing a new class of FMDV inhibitors.

While our study establishes (-)-asarinin as a potent and selective *in vitro* inhibitor, we recognize that the crucial next steps involve validating its potential in a more complex physiological setting [39], where key endpoints will include the reduction of viral load in tissues, amelioration of clinical symptoms, and overall survival rates. These efficacy studies must run in parallel with enzyme kinetics and pharmacokinetic assessments to determine the compound’s ADME profile (absorption, distribution, metabolism, and excretion) and with preliminary toxicology studies to ensure a sufficient safety margin. Concurrently, the (-)-asarinin scaffold provides an excellent starting point for structure–activity relationship (SAR) studies aimed at synthetically optimizing its potency and drug-like properties for this advanced preclinical development pipeline.

Beyond progressing towards a therapeutic application, further work is also required to fully characterize the compound’s interaction with the virus. A key priority will be to investigate the potential for viral resistance. This approach is critical, as previous studies have demonstrated that FMDV can develop resistance to 3D^pol^-targeting antivirals like ribavirin through specific amino acid substitutions (e.g., M296I, P44S, and P169S) [40,41]. Therefore, we plan to address this by serially passaging FMDV in cell culture in the presence of escalating concentrations of (-)-asarinin. Sequencing the 3D^pol^ gene of any emergent resistant strains will not only help predict potential clinical challenges but will also provide invaluable confirmation of the compound’s binding site. Finally, to determine the breadth of its utility, it will be essential to test (-)-asarinin’s efficacy against a broader panel of FMDV serotypes, which is critical for its potential development as a wide-spectrum anti-FMDV agent.

## 5. Conclusions

This study identifies the lignan (-)-asarinin as a potent and selective inhibitor of FMDV replication. Through a combination of computational modeling and multifaceted experimental validation, we demonstrate that its antiviral effect is mediated by specific inhibition of the viral 3D^pol^ polymerase, with no activity observed against the 3C protease. Notably, (-)-asarinin exhibited greater antiviral potency than the benchmark drug ribavirin in cell-based assays. These findings establish the lignan scaffold as a promising new chemotype for anti-FMDV drug discovery and position (-)-asarinin as a strong candidate for antiviral drug development, creating valuable complementary strategies for FMDV control.

## Figures and Tables

**Table 1:** Antiviral and inhibitory activity of lignans. The EC50 and IC50 values given with 95% confidence intervals.

Compounds	Antiviral Activity (EC50 ^a^, µM)			Inhibitory Activity (IC50 ^b^, µM)
	FMDV Specific-Positive Cell (IPMA)	Viral Copy Number (RT-qPCR)	Normalized Delta Ct (RT-qPCR)	GFP Signal (FMDV Minigenome)
(-)-Asarinin	15.11 ± 1.18 (13.72 to 16.49) SI ^c^ = 5.99	36.70 ± 1.57 (36.06 to 37.34) SI = 2.46	31.67 ± 1.50 (30.47 to 32.87) SI = 2.86	10.37 ± 1.01 (9.56 to 11.18) SI = 8.72
Sesamin	52.98 ± 1.72 (51.60 to 54.36) SI = 11.41	82.43 ± 1.92 (81.65 to 83.21) SI = 7.33	60.94 ± 1.79 (60.21 to 61.67) SI = 9.92	74.89 ± 1.87 (73.39 to 76.39) SI = 8.07
Ribavirin (drug control)	39.62 ± 1.60 (38.34 to 40.90) SI = 12.62	32.81 ± 1.52 (31.53 to 34.10) SI = 15.24	29.43 ± 1.47 (28.25 to 30.61) SI = 16.99	1.49 ± 0.17 (1.35 to 1.63) SI = 335.57

^a^ EC50: half maximal effective concentration in BHK-21 cells. ^b^ IC50: half maximal inhibitory concentration in BHK-21 cells. ^c^ Selective index (SI = CC50/EC50).

## Data Availability

The original contributions presented in this study are included in the article/Supplementary Materials. Further inquiries can be directed to the corresponding author.

## Supplementary Materials

The following supporting information can be downloaded at: https://www.mdpi.com/article/10.3390/vetsci12100971/s1. Table S1: Oligonucleotide primers used for cDNA of negative strand synthesis and qPCR.; Table S2: Thirty-six lignans from focused docking and their interactions with catalytic residues.; Table S3: ADME/Tox properties of the six selected lignan compounds; Figure S1: Potential six lignans from focus virtual screening and their interactions.; Figure S2: Antiviral activity of selected lignans during (A) pre-viral entry and (B) protective effect using 0.5% crystal violet staining. Scale bars: 200 μm.; Figure S3: Intracellular FMDV 3C^pro^ inhibition assay of (-)-asarinin and sesamin in a dose-dependent manner.

## Abbreviations

3D^pol^	RNA-dependent RNA polymerase
BHK-21 cells	Baby Hamster Kidney-21 cell
CCK-8	Cell Counting Kit-8
DMSO	Dimethyl sulfoxide
FMD	Foot-and-Mouth Disease
FMDV	Foot-and-Mouth Disease Virus
GFP	Green Fluorescent Protein
HEK-293 cells	Human Embryonic Kidney-293 cell
MOI	Multiplicity Of Infection
scFv-Fc	Single-Chain Variable Fragment fused to the Fc region
TCID50	Half Tissue Culture Infective Dose
