# Supplementary material for: Antiviral Efficacy of Lignan Derivatives (-)-Asarinin and Sesamin Against Foot-and-Mouth Disease Virus by Targeting RNA-Dependent RNA Polymerase (3Dpol)
Source: Vet Sci. 2025 Oct 10;12(10):971. doi: 10.3390/vetsci12100971 (PMC12568301; doi:10.3390/vetsci12100971)
Supplement: Supplementary file 1 [file vetsci-12-00971-s001.zip › vetsci-3898453-supplementary.pdf]

Supplementary materials

Table S1. Oligonucleotide primers used for cDNA of negative strand synthesis and qPCR

Primers	Sequences (5′-3′)	Target region
Gene-specific primer	AAGGGTTGATTGTTGACA	3D gene [1,2]
FMDV-5′UTR_F	CTGTTGCTTCGTAGCGGAGC	5′UTR [1,2,3]
FMDV-5′UTR_R	TCGCGTGTTACCTCGGGGTACC	
FMDV-3D_F	TAGAGCAGTAGATGTTG	3D gene [1,2,3]
FMDV-3D_R	ATGAACATCATGTTTGAGG	

Table S2. Thirty-six lignans from focused docking and their interactions with catalytic residues

	Lignans	Binding Affinity [kcal/mol]	Protein-ligand interaction			
			Asp 240	Asp 245	Asp 338	Asp 339
1	**Eleutheroside E**	-8.8	CH	-	P	H
2	**(-)-Asarinin**	-8.7	-	P	-	-
3	**paulownin**	-8.4	-	CH	-	-
4	**Picropodophyllotoxin**	-8.3	-	CH	CH	-
5	**Sesamin**	-8.1	-	P	-	-
6	**Gomisin A**	-8	CH	-	CH	-
7	Pinoresinol diglucoside	-7.9	H	-	CH	P
8	Acanthoside B	-7.9	-	-	P	H
9	Schisandrol B	-7.9	-	CH	-	-
10	Lappaol C	-7.9	-	H	CH	-
11	Nortrachelogenin-5'-C-beta-glucoside	-7.9	-	H	CH	-
12	Piperitol	-7.8	-	P	-	-
13	(-)-Syringaresnol-4-O-β-D-apiofuranosyl-(1→2)-β-D-glucopyranoside	-7.8	H	-	CH	-
14	Styraxlignolide F	-7.7	-	H	H	-
15	(-)-Epipinoresinol	-7.7	-	CH	-	-
16	Epipodophyllotoxin	-7.7	-	CH	-	-
17	(-)-Licarin B	-7.6	-	CH	P	-
18	Gomisin M2	-7.6	-	H	-	-

19	Magnolignan C	-7.6	-	H	H	-
20	Clemastanin B	-7.5	-	CH	H	-
21	(-)-Holostyligone	-7.5	-	H	CH	-
22	Tracheloside	-7.4	-	P	H	-
23	Veraguensin	-7.4	-	-	CH	-
24	Anhydrosecoisolariciresinol	-7.4	-	P	-	-
25	Pinoresinol	-7.3	-	-	P	-
26	(-)-Pinoresinol	-7.3	-	P	CH	-
27	Podophyllol	-7.3	-	H	-	-
28	(+)-Syringaresinol	-7.2	-	-	CH	-
29	Galgravin	-7.2	-	P	-	-
30	Magnolin	-7.1	-	-	CH	-
31	(+)-Eudesmin	-7.1	-	P	-	-
32	Gomisin G	-7	-	-	H	H
33	(+)-Lyoniresinol 9'-O-glucoside	-7	-	CH	H	-
34	Lariciresinol	-7	-	P	H	-
35	(-)-Lyoniresinol	-7	-	H	CH	-
36	Icariside E5	-7	-	H	H	-

Bold letters of compounds were selected for cell-based assay. CH = Carbon-hydrogen bond; H = Hydrogen bond; P = π-anion

Table S3. ADME/Tox properties of the six selected lignan compounds.

Name	Molecular formulas	MW (Dalton, Da)	cLogP	H-acceptors	H-donors	Total surface area (Angstrom, Å)	Drug-likeness	Mutagenic	Tumorigenic	Reproductive Effective	Irritant
(-)-Asarinin	C20H18O6	354.35	3.2246	6	0	244.06	-1.0557	None	None	None	None
Sesamin	C20H18O6	354.35	3.2246	6	0	244.06	-1.0557	None	None	None	None
Paulownin	C20H18O7	370.35	2.4012	7	1	248.32	0.1606	None	None	None	None
Eleutheroside E	C34H46O18	742.72	-1.9482	18	8	508.9	-4.4264	None	None	None	None
Picropodophyllotoxin	C22H22O8	414.41	1.7928	8	1	290.92	0.17227	None	None	High	None
Gomisin A	C23H28O7	416.46	4.1779	7	1	310.12	3.1219	None	None	None	None

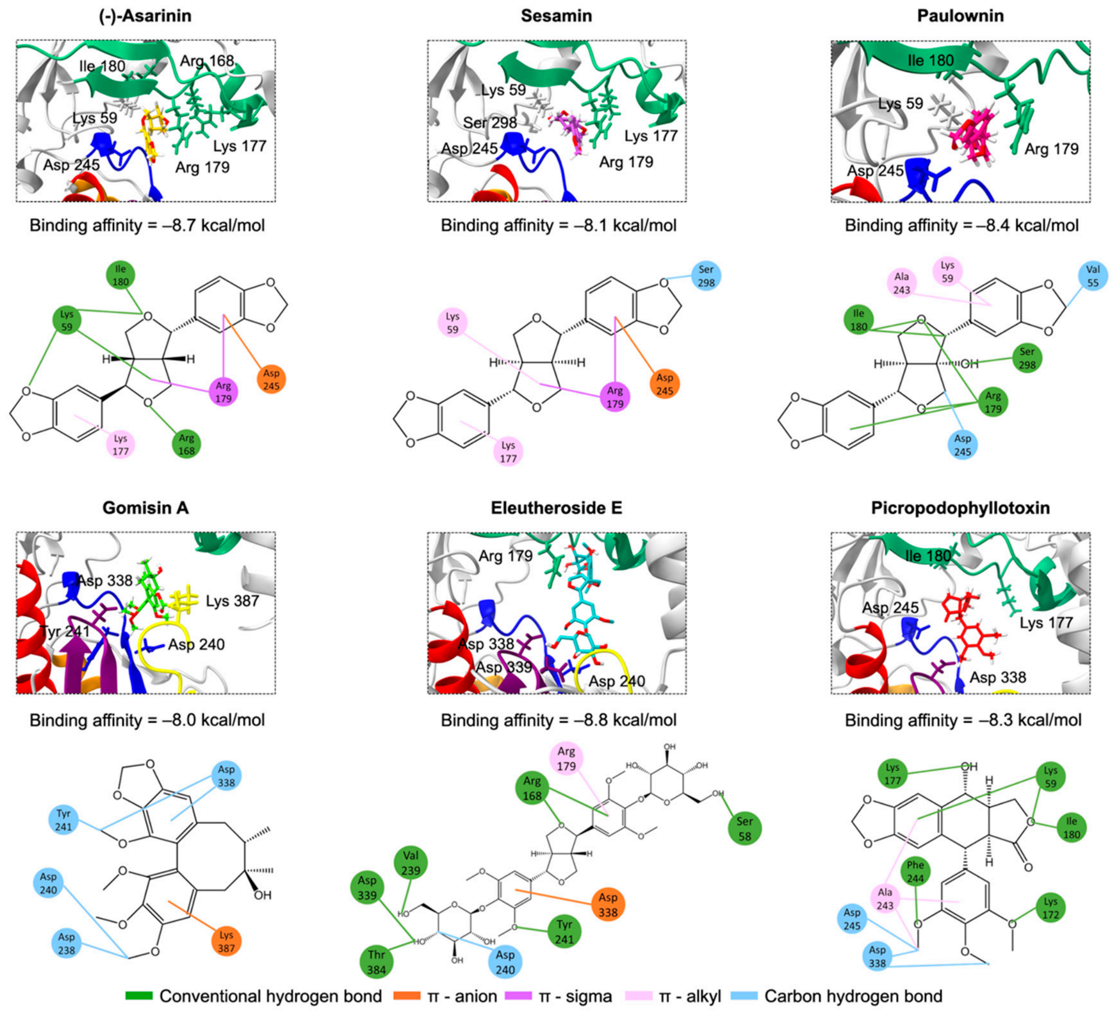

Figure S1. Potential six lignans from focus virtual screening and their interactions.

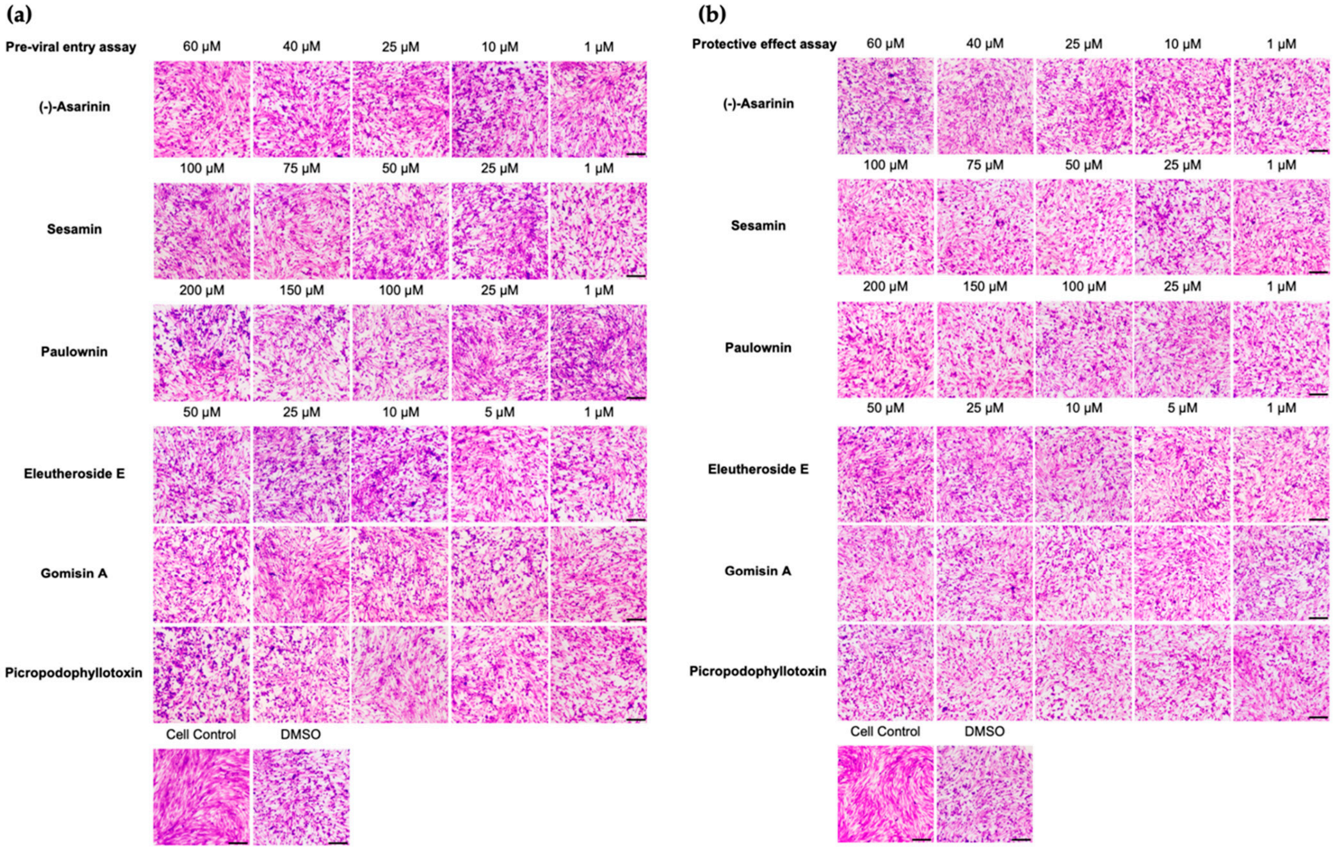

Figure S2. Antiviral activity of selected lignans during (A) pre-viral entry and (B) protective effect using 0.5% crystal violet staining. Scale bars: 200 μm.

A)

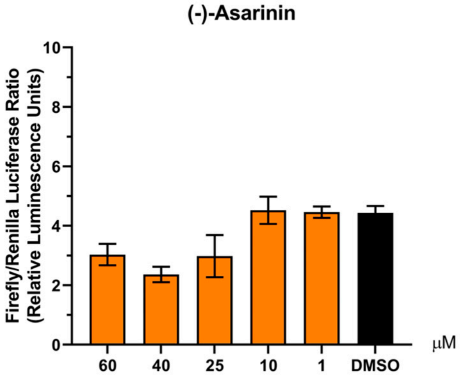

(-)-Asarinin (µM)	Fluc/Rluc ratio	% inhibition
**60**	3.03	-27.51
**40**	2.36	-46.44
**25**	2.98	-32.18
**10**	4.52	2.38
**1**	4.46	0.77
**pBV_3ABCD**	4.43	0
**pBV_mu3ABCD**	149.99	
**VP16**	764.32	
**mock**	1.57	

B)

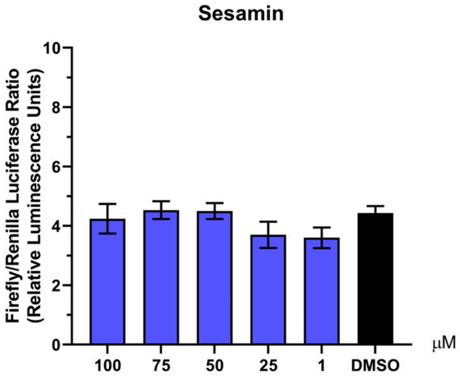

Sesamin (µM)	Fluc/Rluc ratio	% inhibition
**100**	4.24	-5.02
**75**	4.53	2.63
**50**	4.50	2.02
**25**	3.70	-15.37
**1**	3.60	-18.44
**pBV_3ABCD**	4.43	0
**pBV_mu3ABCD**	149.99	
**VP16**	764.32	
**mock**	1.57	

C)

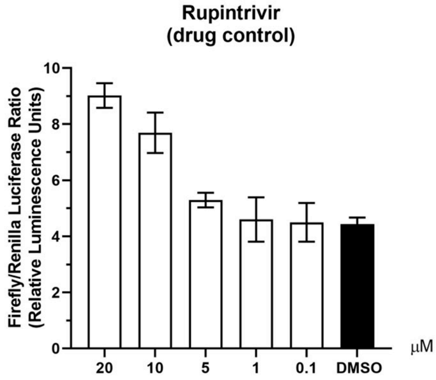

Rupintrivir (µM)	Fluc/Rluc ratio	% inhibition
**20**	9.02	103.58
**10**	7.69	73.35
**5**	5.29	19.76
**1**	4.60	3.91
**0.1**	4.50	2.26
**pBV_3ABCD**	4.43	0
**pBV_mu3ABCD**	149.99	
**VP16**	764.32	
**mock**	1.57	

Figure S3. Intracellular FMDV 3C^{pro} inhibition assay of (-)-asarinin and sesamin in a dose-dependent manner.

References

1. Theerawatanasirikul, S.; Semkum, P.; Lueangaramkul, V.; Chankeeree, P.; Thangthamniyom, N.; Lekcharoensuk, P. Non-nucleoside inhibitors decrease foot-and-mouth disease virus replication by blocking the viral 3Dpol. *Viruses* **2023**, *15*, 124, doi:10.3390/v15010124.
2. Mana, N.; Theerawatanasirikul, S.; Semkum, P.; Lekcharoensuk, P. Naturally derived terpenoids targeting the 3dpol of foot-and-mouth disease virus: An integrated in silico and in vitro investigation. *Viruses* **2024**, *16*, 1128, doi:10.3390/v16071128.
3. Semkum, P.; Thangthamniyom, N.; Chankeeree, P.; Keawborisuth, C.; Theerawatanasirikul, S.; Lekcharoensuk, P. The application of the gibson assembly method in the production of two pKLS3 vector-derived infectious clones of foot-and-mouth disease virus. *Vaccines* **2023**, *11*, 1111, doi:10.3390/vaccines11061111.
